# The Effects of Maternal Supplementation of Polyunsaturated Fatty Acids on Visual, Neurobehavioural, and Developmental Outcomes of the Child: A Systematic Review of the Randomized Trials

**DOI:** 10.1155/2012/591531

**Published:** 2012-01-18

**Authors:** Andrea Lo, Julianna Sienna, Eva Mamak, Nada Djokanovic, Carol Westall, Gideon Koren

**Affiliations:** ^1^Motherisk Program, Division of Clinical Pharmacology and Toxicology, The Hospital for Sick Children, University of Toronto, 555 University Avenue, Toronto, ON, Canada M5G 1X8; ^2^Department of Ophthalmology and Vision Sciences, The Hospital for Sick Children and University of Toronto, 555 University Avenue, Toronto, ON, Canada M5G 1X8; ^3^Research Insttitute, The Hospital for Sick Children, University of Toronto, 555 University Avenue, Toronto, ON, Canada M5G 1X8

## Abstract

Polyunsaturated fatty acid (PUFA) use in pregnancy has been promoted as beneficial for visual and neurobehavioural development in the fetus. However, no systematic review of the randomized trials has been conducted. The objective of this review was to evaluate potential advantages of this regiment by reviewing all randomized trials in pregnancy. *Methods*. Systematic review of randomized controlled studies comparing cognitive and visual achievements among infants whose mothers were treated and untreated with PUFA during gestation. *Results*. Nine studies met the inclusion criteria, three focusing on visual and six on neurobehavioural development. Due to differing outcome measurements in the infants, the studies could not be combined into a formal meta-analysis. Synthesizing the existing data, for both visual and neurobehavioural development, most studies could not show sustained benefits to infant cognition or visual development. *Conclusion*. At the present time a recommendation to change practice and supplement all expecting mothers with PUFA to improve offspring vision or neurobehavioural function is not supported by existing evidence.

## 1. Introduction

Polyunsaturated fatty acids (PUFAs) of the *ω*-3 and *ω*-6 families cannot be synthesized by the human body [1], making the parent fatty acids of these families—alpha-linolenic acid (ALA) and linoleic acid (LA)—essential fatty acids that must be obtained from the diet [2]. ALA is converted into eicosapentaenoic acid (EPA) and then to docosahexaenoic acid (DHA), a critical component of cell membranes especially in the brain and retina. LA is converted into arachidonic acid (AA), a membrane component and a precursor to signaling molecules [2]. The ratio of the *ω*-3 to *ω*-6 families of PUFAs is critical because both families are metabolized by the same enzymes, and increasing the amount of *ω*-3 fatty acids (FAs) in the diet, for example, may decrease the availability of the *ω*-6 products. Therefore, there is a potential risk of reducing AA levels in the fetus with maternal supplementation of *ω*-3 FAs [1].

 Because the PUFAs required by the fetus are supplied by preferential placental transfer of preformed long-chain PUFA (LC-PUFA) rather than the precursors ALA and LA, it has been proposed that additional maternal supply of DHA and AA during pregnancy may improve early cognitive and visual development [3]. The limited available data on LC-PUFA in the developing human brain indicates that fetal accumulation of LC-PUFA is slow in the earlier weeks of gestation and rapidly increases in the third trimester [4].

 Among the *ω*-3 FAs subtypes, DHA is the only one that accumulates to an appreciable extent in the developing brain and eye [5]. DHA is actively and preferentially transferred to the fetus by specific fatty acid placental transfer and membrane binding proteins. Of all cells, the highest content of DHA is found in retinal photoreceptors, the cells responsible for phototransduction [6]. As well, the visual cortex of the brain has high levels of DHA. Brain and visual development is most sensitive to malnutrition in the third trimester and in the first 18 weeks of postnatal life. In Rhesus monkeys and rats fed diets limited in LCPUFA during pregnancy, there are reduced levels of PUFAs in pups in both the retina and the visual cortex [7–9]. Pregnant Rhesus monkeys and their pups fed diets low in n-3 fatty acids exhibit below normal visual acuity scores at 4–12 weeks compared to mother and infant monkey pairs fed diets with “ample” n-3 fatty acids [9].

 In animal studies, severe restriction of *ω*-3 fatty acids results in lower concentrations of DHA in the brain and poorer cognitive and behavioural capacities [10]. Several human studies have suggested that maternal diet rich in seafood correlates with higher scores on tests of cognitive function. Observational studies have suggested that prenatal AA status correlates positively with neurodevelopmental outcome during early infancy, but not at older ages [4].

 In Health Canada “Prenatal Nutrition Guidelines for Health Professionals,” women are advised to consume at least 150 g of cooked fish weekly, preferably those with lower levels of contaminants such as methyl mercury and polychlorinated biphenyls (PCBs), suggesting that fish intake during pregnancy may be linked to better infant and child development [11]. With respect to LC-PUFA supplementation, it is advised that fish oil supplements should not be considered equivalent to eating fish, and though they provide *ω*-3 fatty acids, there is insufficient evidence to draw conclusions on the effects of fish oil supplementation on infant development [12]. Observational studies correlating PUFA with fetal development suffer from numerous confounders that may affect outcome, such as socioeconomic status, maternal education, and other nutrients status.

 The objective of this systemic review was to evaluate the potential effects of interventional supplementation of *ω*-3 FAs during the pregnancy period only on infant neurobehavioral and visual development, without the potential effects of breastfeeding or dietary supplementation.

## 2. Material and Methods

### 2.1. Inclusion and Exclusion Criteria

Studies included in this review were randomized control trials (RCTs) comparing LC-PUFA supplementation with placebo or no supplementation in pregnant women. Trials that used supplementation only during breastfeeding and/or infant dietary supplementation were excluded. In contrast, trials that started in pregnancy but continued during breastfeeding were included. Trials reporting only biochemical outcomes or using animals were not included and only original research articles were considered. Trials in which precursors of essential FAs (ALA and LA) were used in intervention group were not included because the preferential placental transfer for LC-PUFAs precursors is far less effective. Abstracts for which a published full paper could not be located were excluded for this review.

 To ensure a high quality of evidence, we restricted the review to RCTs with Jadad scores of 3 or greater on the 5-point scale [13].

### 2.2. Search Strategy

We completed a computerized literature search of MEDLINE (1950–June 2010), EMBASE (2010), the Cumulative Index to Nursing and Allied Health (CINALH) (from inception to June 2010), and the Cochrane Library (2010). We supplemented this search by investigating relevant references from published reviews [2–4, 14, 15]. There was no limit on the language of publication.

 Search terms used were “omega or n-6 or n-3 or eicosapentaenoic acid or EPA or docosahexaenoic acid or DHA or arachidonic acid or LC-PUFA or long-chain fatty acid or essential fatty acid or fish oil or fatty acid” and “supplementation” and “pregnancy or maternal.”

### 2.3. Methods of Review

**Trial Selection**

Two researchers independently applied the inclusion criteria to each potential relevant trial and differences with regards to their eligibility were resolved by consensus.

### 2.4. Quality Assessment

Two researchers independently assessed the quality of the studies that met the inclusion criteria using the Jadad method [13], and articles included had to score between 3 and 5 on the 5-point scale.

## 3. Results

**Figure 1:**
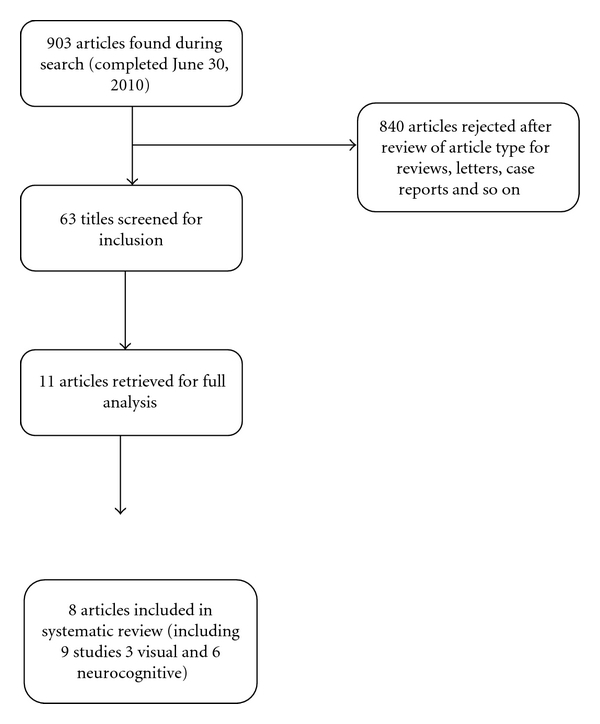
Search strategy flow chart.

All 9 identified randomized trials met our inclusion criteria, and all of them achieved at least 3 on the Jadad score (Figure 1). Three trials were focused on retinal development [16–18] while six studied neurodevelopment [16, 19–23]. Though trials with all LC-PUFA (*ω*-3 and *ω*-6) supplementation were considered for this review, none of the trials used *ω*-6 in the intervention group. The characteristics of the included trials are summarized in Table 1. The duration, sources, and amounts of *ω*-3 LC-PUFA, DHA, and EPA supplied varied among trials. The doses ranged from 2 × 100 mg DHA/week [17, 18] to 1.1 g EPA, 2.2 g DHA/day, which were used in the study by Dunstan et al. [19]. The trials differed in the starting point of intervention, ranging from the 15th [17, 18] to the 25th weeks of gestation [23]. All trials ended supplementation at delivery, with the exception of the studies by Helland et al. [20–22], which continued supplementation until 3 months after delivery. Because these studies did not change the practice of breastfeeding between control and treatment groups, we still included them in our review. Because the methods of measuring visual and neurodevelopmental outcomes varied widely among studies, the combination of the results into a formal meta-analysis was deemed inappropriate.

### 3.1. Effectiveness of PUFA for Visual Development

To allow readers who are not specialists in measuring visual development to follow the results, the description of the study results is preceded by description of the methods used by the different groups. There are various ways to assess vision in humans; however, not all of these methods can be used as diagnostic tools in children, let alone in infants. Both visual function (the ability to see) and visual acuity (a quantifiable measure of vision function) can be tested. There are several aspects of visual acuity (the spatial limit of visual discrimination): detection, resolution, identification, and hyperacuity. A commonly used test, Teller Acuity Cards [25], tests resolution, or the smallest angular separation between two objects side by side [26]. 

 Visual function may also be assessed using objective electrophysiological measures such as the visual evoked potentials (VEPs) (steady state and transient). Retinal function can be assessed using the electroretinogram (ERG). The International Society of the Clinical Electrophysiology of Vision (ISCEV) provides standards for both VEP and ERG testing [27, 28]. VEPs are recorded with electrodes placed on the back of the scalp to measure the responses generated by the visual cortex in response to a change in visual stimulus. Responses to this test indicate the function of the visual pathway: retina, optic nerve, and brain (specifically the occipital cortex) and are dependent on unobstructed ocular media such as the cornea and lens. When used to assess visual function, the VEP does not localize where in the visual pathway damage exists. ERGs are measured using electrodes placed on the cornea of the eye and forehead. Subjects are presented with flashes of light of different intensities and the resulting responses generate characteristic outputs. The positive and negative peaks of different outputs are known to originate in specific areas of the retina which localize damage to a specific layer of the retina. Both VEPs and ERGs can be described in terms of amplitude and implicit time or latency of the response.

 Judge et al. conducted a longitudinal, double-blinded RCT of thirty nonsmoking women supplemented with either DHA-rich (mean = 214 mg/d) cereal bars or placebo bars starting at 24 weeks of gestation [24]. Infants were assessed at 4 and 6 months of age by Teller Acuity Cards Procedure (ACP) which is a type of preferential looking test for resolution. This technique assumes that children would rather look at a pattern than a blank stimulus [29]. It consists of a series of gray cards with one circle with a black and white grating (of different frequencies) and another card of equal luminance to the grating. An observer must identify that the child has preferentially looked at the grated stimulus (but are themselves blinded to which side the grated stimulus is on). The test is repeated, switching the side of the grated stimulus, until the observer feels they can reliably decipher whether the child is in fact preferentially looking at the grated card. That grating frequency, referred to as a spatial frequency, is then said to be above the child's acuity threshold. After adjusting for potential confounding factors, including infant feeding type, there was a significant difference in visual acuity between groups at 4 months, but not at 6 months postnatally. Both supplemented and nonsupplemented groups showed an increase in ACP score over time but the change in score over time was not significantly different between the two groups and the authors suggested that DHA supplementation aided in visual system maturation [24].

 Malcolm et al. conducted a prospective placebo controlled, randomized double-blind trial investigating electroretinogram (ERGs) in infants born to 100 woman supplemented with 200 mg DHA or 200 mg sunflower oil placebo from week 15 of pregnancy until birth [17]. This study was well designed and used the bipolar Burien Allen electrodes and a Ganzfeld dome, known to elicit repeatable valid responses [28]. These tests were administered to the children without sedation which can be problematic based on the child's level of cooperation. The authors reported that infant DHA status, and ERG implicit times, amplitude, and stimulus response functions at birth, did not differ between groups for 60 infants tested within one week of birth [17]. However, infants in the highest quartile for cord blood DHA had significantly higher retinal sensitivity (log⁡⁡*σ*) as compared with those in the lowest quartile, and those in the highest quartile for plasma DHA were born at significantly later gestational age than those in the lower quartile, regardless of maternal supplementation type. The authors concluded that, although maternal supplementation had no effect on infant DHA status or retinal development, those infants with higher DHA status had increased retinal sensitivity and longer gestational age [17].

 Malcolm et al. also tested this cohort using VEPs [18]. After supplementation, red blood cell (RBC) DHA concentration and the percentage of total fatty acids (%TFA) in pregnant women (*n* = 54) were higher in the fish oil group than in the placebo group from 28 wks to delivery (*P* < 0.05). As before, DHA supplementation did not significantly elevate levels of DHA measured as RBC concentration of %TFA in umbilical cord blood. Fifty-five infants tested showed no significant group differences in mean peak latencies of major components of flash VEP waveform or in the peak latency of the P100 component of the pattern-reversal VEP, and no significant correlation was detected between flash VEP peak latencies and RBC/plasma DHA levels in cord blood at any time (birth, 50 weeks after conceptional age (PCA), and 66 weeks PCA). Similarly, no differences were found in the threshold check size of the pattern-reversal VEP at 50 or 66 weeks PCA between supplementation groups. Pattern-reversal VEP maturity, measured as shorter peak latency, correlated at 50 weeks and 66 weeks PCA with cord DHA status, but not with maternal supplementation group. Here, infants in the top quartile of RBC DHA status (median −5.46%) did not differ significantly in VEP maturity from those in the lowest quartile (median −3.45%).

### 3.2. Neurodevelopment

Six trials met the inclusion criteria, achieving Jadad scores of at least 3. Of these six trials, three were published by Helland et al. based on followup of one RCT [20–22]. Therefore, there was a total of four independent RCTs included in this part of the systemic review.

 Various methods of measuring neurodevelopment were used in the six papers accepted for this review, and these will be described to allow the reader to evaluate the results. The Bayley Scales of Infant Development Second Edition (BSID-II) is a standardized test used to assess motor and cognitive development of children between the ages of zero and three years. Raw scores are compared with age-based normative data to determine individual standard scores. The BSID-II includes two subscales: the mental development index (MDI) and psychomotor development index (PDI) [30, 31]. Using this method, Tofail et al. found no significant difference between the MDI and PDI scores of 10-month-old children of mothers supplemented with fish oil or soy oil during the last trimester of pregnancy [23]. This study took place in Bangladesh, and 28% of the mothers in the test population suffered from undernutrition. The authors speculated that because there was attrition of 38% from the original randomized sample and the attrition sample was at greater risk for neurodevelopmental problems due to clinical difficulties during pregnancy, the lack of treatment effect may be attributed to the attrition of those that likely would have benefited most from treatment. In addition, the authors commented on the lack of validation or standardization of the Bayley-II in Bangladesh, which may have limited the sensitivity to detect minor differences between groups [23].

 Judge et al. used the infant planning test and Fagan tests to compare the neurodevelopment of 9-month-old infants of mothers supplemented with DHA or placebo during pregnancy [16]. The Fagan test is used to estimate infants' recognition memory, as a proxy of intellectual ability, by presenting them with novel and familiar facial pictures [32]. Those children who spend more time fixating on novel stimuli are given higher scores. This test is thought to represent the speed with which infants acquire new knowledge and has been shown to moderately correlate with IQ at 2 years of life [33]. This task assesses a single aspect of development, namely, facial (visual) recognition.

 Other standardized infant developmental tests (e.g., Bayley Scales of Infant Development, Mullen Scales of Early Learning) use a variety of tasks to estimate infant IQ and, based on their assessment of other functions (e.g., motor, language, and other problem-solving tasks), may act as a better estimate of later cognitive functioning. The infant planning test requires infants to execute a series of steps to retrieve a toy as a measure of their problem solving ability [33–37]. This test, as well as its method for scoring and assessing performance, is unpublished and has no available reliability or validity information, limiting the interpretation of the findings. The women from both study groups were instructed to consume 3, 5, or 7 cereal bars/wk from week 24 to delivery. The average was 5 bars a week, averaging 214 mg/d of DHA consumption. Significantly higher problem-solving scores (i.e., better performance) were associated with maternal PUFA supplementation as measured by the infant planning test. However, there was no difference detected in facial recognition by the Fagan test. The authors stated that the lack of significant differences may not be surprising, as the Fagan test is more sensitive at 4 and 6 months of age [38]. Another possibility is that PUFA supplementation may not impact this specific skill of development as it is thought to be a measure of selective visual attention and facial recognition.

 Helland et al.'s three studies examined infants of mothers supplemented daily with 10 mL cod liver oil (1183 mg DHA, 803 mgEPA) or 10 mL corn oil placebo, from wk 18 of pregnancy until 3 months after delivery, to measure effects on infant neurodevelopment [20–22]. 242 of the 251 infants were breastfed at least until 3 months of age, and a subset (*n* = 130) were started on supplement of cod liver oil (5 mL daily from 4 wks of age). No differences were found between the infant diet in the supplemented groups in terms of PUFA content, and for the sake of the present systematic review we were interested only in groups that differed with respect to maternal supplementation. Helland et al. measured neurodevelopment at ages day 2 and 3 months using EEG (*n* = 149) [20], 6 and 9 months using the Fagan test (*n* = 245) [21], and 4 years (*n* = 84) and 7 years (*n* = 143) using the Kaufman Assessment Battery for Children (K-ABC) [22], in which sequential processing, simultaneous processing, achievement, and nonverbal abilities are scored in a multi-subtest battery. The raw scores for each category were converted to standard scores according to the American norms since the Norwegian version has not been standardized. There were no differences found in the test scores at any of the ages [20–22], except at 4 yrs, when children of cod-oil-supplemented mothers scored higher on the mental processing composite of K-ABC [22].

 Dunstan et al. studied the effects of fish oil (3.7 g of *ω*-3 PUFA/d) supplementation during pregnancy from wk 20 to delivery on several scales of cognitive assessment of the child at age 2.5 yrs [19]. The Griffiths Mental Development Scales (GMDS) used by the group have six subscales of development (locomotor, personal, social, speech and hearing, eye and hand coordination, and performance and practical reasoning) and a general score is derived from the averages of the subscale scores [39]. The Peabody Picture Vocabulary Test (PPVT) III is a test of English receptive vocabulary [40]. Finally, the Child Behaviour Checklist (CBCL) measures parental perception of child competencies, behaviours, and language development [41]. The study used all three measures at age 2.5−3 yrs and found that the infants of the supplemented group had significantly higher scores on the eye and hand coordination subtest of the GMDS than those of the control group (*P* = 0.02). However, there were no significant differences in the scores for any other sections of the GMDS, the PPVT-III, or the CBCL. The relatively high doses of *ω*-3 PUFA supplementation in this study were not associated with any deleterious effects on neurodevelopment or growth [19].

## 4. Discussion

The 8 randomized trials reviewed by us focusing on the effects of maternal PUFA supplementation on the neurocognitive and retinal development in the child have found very limited, if any, benefits to supplementation. Even in the studies that found statistically significant differences between treatment and control groups, the differences were small and of little potential clinical importance. These trials have found that even high doses of supplementation of *ω*-3 PUFA (up to 3.7 g/d) were not associated with any detrimental effects [19].

 These studies did not detect a relationship between the doses of supplementation and measured effects [18, 19] and found that, although there was an association between infant DHA status and retinal/VEP maturity, there was no correlation between maternal supplementation of PUFAs and infant DHA status [18, 19]. In contrast, Dunstan et al., using the highest doses of 4 × 1g fish oil/day (3.7 g DHA/d), found improved scores only in the hand and eye coordination subtest in the K-ABC for infants in the treatment group [19]. Although the study by Judge and colleagues was well designed, the sample size was small and the vehicle of DHA administration (DHA-rich cereal bars) differed from the other studies on visual development which used fish oil capsules.

Measurements of maternal or infant DHA status were not reported. The study did not report whether acuity was tested monocularly or binocularly. As effects were found at 4 months but not 6 months, it is possible that maternal supplementation provides an initial advantage which disappears with time. One would only expect a continued difference if prenatal supplementation predisposed infants to have better vision. Two different and inconsistent values for visual acuity scores were presented in the abstract versus the text. Contact with the authors clarified that the value in the abstract was based on using the GLM model (a statistical method of using least squares to fit general linear models), whereas the value in the text was calculated using group means.

 The detected difference of 0.5 cycles/degree (c/d) (see Table 1), although statistically significant, falls within the normal range for age (binocular acuity) at four months (6.8–1.7 c/d) and six months (9.1–2.2 c/d) [25]. The authors noted that a limitation of this study was in the use of ACP, which, although shown to be repeatable [30, 31], has inherent subjectivity.

 As in the ERG study by Malcolm [17], maternal supplementation did not correlate with infant DHA status [17]. This is interesting especially because the mothers in each group did have significantly different levels of DHA and %TFA at delivery. This study used solid methodology to test VEP responses, with the authors conducting both flash and transient pattern-reversal VEPs. The flash VEP is only able to give an indication of whether the visual cortex responds to light stimulation, whereas the pattern-reversal VEP yields information on the quality of the response. This may explain why significant differences were found in pattern-reversal VEPs and not in flash VEPs. Sweep VEPs are likely the best way to test cortical responses in infants, because they take the shortest time to elicit and there would be less chance of a child loosing attention to the stimulus [42–44]. The authors reported VEP results using latencies which are less variable than amplitudes [44]. The authors of this study reported averaging their trials over 30–50 epochs, which is certainly sufficient to minimize the signal-to-noise ratio.

 The results of both studies conducted by Malcolm et al. suggest that infant DHA status, but not maternal supplementation, is correlated with infant visual development. Synthesizing these three studies, at the present time a recommendation to change practice and supplement all expecting mothers with PUFA to improve offspring vision is not supported by the existing evidence.

 The data of the studies included in this systematic review could not be combined into a formal meta-analysis because the measures used in the studies varied greatly with regard to dosage, length of supplementation, age of testing, and the measures of effect. Even without quantitatively combining the results, it is evident that any beneficial effect from *ω*-3 PUFA supplementation, if it exists, is very small and therefore likely not clinically significant. Cohen et al. conducted an analysis on prenatal intake of n-3 PUFAs and cognitive development, in which they estimated that increasing maternal DHA intake by 1 g/d may increase child IQ by about 1.3 points.

 In explaining the results of the neurocognitive interventions, Helland and colleagues suggested that the lack of measured effect may be because the effects of the *ω*-3 PUFAs are diluted by several other factors such as other nutrients, drugs, social stimulation, and diseases. Other possible explanations were lack of effect of *ω*-3 PUFAs or that their methods of cognitive testing were not sufficiently sensitive to detect differences at these ages. Alternately, performance on the specific visual task used in the Fagan test was not impacted [20], as found in the Judge et al. study [16]. In contrast, early neurodevelopment, when assessed with a measure inclusive of a broader range of skills (K-ABC), was sensitive to these changes. Even when group differences in mental processing scores were detected at four years of age, these were not of a magnitude to make a significant clinical impact (4 IQ points, or about one-quarter of a standard deviation).

 In synthesizing the existing neurocognitive studies, the papers included in this systematic review have yielded variable results in terms of whether PUFA supplementation during pregnancy was of benefit to infant neurocognitive development. In the longitudinal study by Helland et al., the limited effects evident at four years of age were nullified three years later [20–22]. In Dunstan et al.'s study, a single positive effect was contrasted by mostly negative results [19]. When comparing numerous endpoints, a single positive result may arise by chance only, as *P* < 0.05 means a 1 in 20 chance of “no difference” becoming “significant,” especially since multiple comparison correction for the large number of comparisons was not performed in Dunstan et al.'s study [19]. Similarly, Judge et al. found one significantly positive effect in a large number of negative tests [16]. If there was a genuine favorable effect, it was of small magnitude and may not persist in later years [21]. Thus, in considering the results of these six studies, at the present time a recommendation to change practice and supplement all expecting mothers with PUFA to improve infant neurodevelopment is not strongly supported by the existing research results.

 Several limitations exist in the body of knowledge analyzed by us. First, studies that examined the effects of LC-PUFAs in preterm infants were not included in this review. However, developmental benefit to PUFA supplementation may be more consistent in infants born prematurely. It may be argued that as preterm infants are denied the full gestation period to accumulate an adequate amount of DHA, they may benefit the most from increased maternal DHA levels during pregnancy, achieved with DHA supplementation.

 Preterm infants fed with DHA-supplemented formula have shown better visual resolution acuity at 2 and 4 months [45] and higher Bayley mental and psychomotor development scores at 118 weeks [46].

 Second, the included studies have employed a variety of tests in order to measure neurocognitive and retinal development. Because these measures assess different components of brain development and aspects of cognition, it may not be surprising that there are inconsistent results among different studies.

## 5. Conclusions

Our systematic review of RCTs suggests that the research available to date regarding the maternal supplementation of PUFAs in retinal and neurocognitive development of the infant is not consistent in showing a benefit to supplementation. However, there is evidence that dietary deficiency in LC-PUFAs can adversely affect retinal and neurocognitive development outcomes in animals, and these data are corroborated in nutritionally impaired women in Bangladesh thus, it is important to maintain a healthy diet that contains sufficient sources of PUFAs, such as eggs and fish.

## Figures and Tables

**Table 1:** Description of included studies.

Study (year)	Jadad score	Treatments—maternal diet supplementation	Outcome	*N*	Test results
Treatments and outcomes for studies considered—retinal development					

Malcolm [17]	3	*Controls* (C): 2 placebo capsules (323 mg sunflower oil)/day *Treatment*: 2 fish oil capsules (blended fish oil, Marinol D40: 100 mg DHA)/day From wk 15 of pregnancy until delivery	Age: 50 and 66 wks after conceptional age—*visual evoked potential * ** (**transient VEP P100 peak latencies (ms))	DHA: *n* = 31 C: *n* = 29	No effect of group at any time
Judge [16, 24]	4	*Controls*: 3,5, or 7 placebo cereal bars (corn oil)/wk *Treatment*: 3, 5, or 7 cereal bars (300 mg DHA)/wk (average was 5 bars/wk: 214 mg/d of DHA) From wk 24 of pregnancy until delivery	Age: 4 and 6 mo *Teller Acuity Cards *	DHA: *n* = 16 C: *n* = 14	4 months DHA: 3.7 ± 1.3 c/d C: 3.2 ± 1.3 c/d *P* = 0.018 No difference at 6 months
Malcolm [18]	4	*Controls*: 2 placebo capsules (sunflower oil)/day		DHA: *n* = 31	
		*Treatment*: 2 fish oil capsules (Marinol D40: 100 mg DHA)/day, total 200 mg/d from wk 15 of pregnancy until delivery	Age: within 1 wk of birth *Scotopic electroretinogram *	C: *n* = 29	No effect of group on VEP maturity

Treatments and outcomes for studies considered—neurodevelopment					

Tofail [23]	4	*Controls*: 4 placebo capsules (soybean oil: total 2.25 g LA and 0.27 g LNA)/day *Treatment*: 4 fish oil capsules (total 1.2 g DHA and 1.8 g EPA)/day From wk 25 of pregnancy until delivery	Age: 10 mo *Bayley Scales of Infant Development II, * *Mental Developmental Index (MDI) * *Psychomotor Developmental Index (PDI) *	Fish oil *n* = 125 Soy oil *n* = 124	Mental developmental index: Fish 102.5 (8.0) Soy 101.5 (7.8) 95% CI of difference between means: −0.98, 3.0 Psychomotor developmental index Fish 101.7 (10.9) Soy 100.5 (10.1) 95% CI of difference between means: −1.3, 3.8
Judge [16, 24]	3	*Controls*: 3, 5, or 7 placebo cereal bars (corn oil)/wk *Treatment*: 3,5, or 7 cereal bars (300 mg DHA)/wk (overall average of 214 mg/d of DHA) From wk 24 of pregnancy until delivery	Age: 9 mo *Infant planning test (IPT) * *Fagan test of infant intelligence (FTII) *	IPT DHA: *n* = 14 C: *n* = 15 FTII DHA: *n* = 15 C: *n* = 15	Infant planning test (1) Intention score DHA = 8 (2.3), C = 6.7 (3) *P* ^2^ = 0.017 (2) Intentional solutions DHA = 2.5 (1.3), C = 1.7 (1.5) *P* ^2^ = 0.011 Fagan test Scores for 5 different variables, no significant difference in any
Helland [20–22]	4	*Controls*: 10 ml corn oil (4747 mg LA, 92 mg alpha LA)/day *Treatment*: 10 ml cod liver oil (1183 mg DHA, 803 mg EPA)/day From wk 17–19 of pregnancy until *3 months after* delivery	Age: 2nd day and 3 mo *EEG * Age: 6 mo and 9 mo: *Fagan test *	2nd day EEG T: *n* = 66 C: *n* = 83 3 mo T: *n* = 61 C: *n* = 61 Fagan 6 mo T: *n* = 144 C: *n* = 118 9 mo: T: *n* = 130 C: *n* = 115	EEG: no difference b/w groups at both ages. Fagan: no difference at either time.
Helland [20–22]	4	*Controls*: 10 ml corn oil (4747 mg LA, 92 mg alpha LA)/day *Treatment*: 10 ml cod liver oil (1183 mg DHA, 803 mg EPA)/day From wk 18 of pregnancy until *3 months* after delivery	Age: 4 yrs *Kaufman Assessment Battery for Children (K-ABC) *	Cod oil: *n* = 48 Corn oil: *n* = 36	K-ABC mental processing composite 4 yrs: 106.4 (7.4) versus 102.3 (11.3) for control *P* = 0.049
Helland [20–22]	3	*Controls*: 10 ml corn oil (4747 mg LA, 92 mg alpha LA)/day *Treatment*: 10 ml cod liver oil (1183 mg DHA, 803 mg EPA)/day From wk 18 of pregnancy until *3 months after* delivery	Age: 7 yrs *Kaufman Assessment Battery for Children (K-ABC) *	Cod: *n* = 82 Corn: *n* = 61	K-ABC mental processing composite—no difference
Dunstan [19]	3	*Controls*: 4–1 g olive oil capsules (total 2.7 g n9 oleic acid)/day *Treatment*: 4–1g fish oil capsules (total 1.1 g EPA, 2.2 g DHA)/day (3.7 g of *ω*-3 PUFA/d) From wk 20 of pregnancy until delivery	Age: 2.5 yr *Griffiths Mental Development Scales (GMDS) * *Peabody picture vocabulary test IIIA * *Child Behavior checklist 1.5–5 y *	T: *n* = 52 C: *n* = 46	Hand and eye coordination T (*n* = 33): 114 (10.2) C (*n* = 39): 108 (11.3) *P* = 0.008 No significant difference on other tests

## References

[1] Dubnov-Raz G, Finkelstein Y, Koren G (2007). *ω*-3 fatty acid supplementation during pregnancy: for mother, baby, or neither?. *Canadian Family Physician*.

[2] Koletzko B, Lien E, Agostoni C (2008). The roles of long-chain polyunsaturated fatty acids in pregnancy, lactation and infancy: review of current knowledge and consensus recommendations. *Journal of Perinatal Medicine*.

[3] Szajewska H, Horvath A, Koletzko B (2006). Effect of n-3 long-chain polyunsaturated fatty acid supplementation of women with low-risk pregnancies on pregnancy outcomes and growth measures at birth: a meta-analysis of randomized controlled trials. *American Journal of Clinical Nutrition*.

[4] Hadders-Algra M (2008). Prenatal long-chain polyunsaturated fatty acid status: the importance of a balanced intake of docosahexaenoic acid and arachidonic acid. *Journal of Perinatal Medicine*.

[5] Martinez M (1992). Tissue levels of polyunsaturated fatty acids during early human development. *Journal of Pediatrics*.

[6] Fliesler SJ, Anderson RE (1983). Chemistry and metabolism of lipids in the vertebrate retina. *Progress in Lipid Research*.

[7] Neuringer M, Connor WE, Lin DS, Barstad L, Luck S (1986). Biochemical and functional effects of prenatal and postnatal omega 3 fatty acid deficiency on retina and brain in rhesus monkeys. *Proceedings of the National Academy of Sciences of the United States of America*.

[8] Benolken RM, Anderson RE, Wheeler TG (1973). Membrane fatty acids associated with the electrical response in visual excitation. *Science*.

[9] Neuringer M, Connor WE (1986). n-3 fatty acids in the brain and retina: evidence for their essentiality. *Nutrition Reviews*.

[10] McCann JC, Ames BN (2005). Is docosahexaenoic acid, an n-3 long-chain polyunsaturated fatty acid, required for development of normal brain function? An overview of evidence from cognitive and behavioral tests in humans and animals. *American Journal of Clinical Nutrition*.

[11] Allain-Doiron A, Gruslin A, Innis SM

[12] Cohen JT, Bellinger DC, Connor WE, Shaywitz BA (2005). A quantitative analysis of prenatal intake of n-3 polyunsaturated fatty acids and cognitive development. *American Journal of Preventive Medicine*.

[13] Jadad AR, Moore RA, Carroll D (1996). Assessing the quality of reports of randomized clinical trials: is blinding necessary?. *Controlled Clinical Trials*.

[14] Ryan AS, Astwood JD, Gautier S, Kuratko CN, Nelson EB, Salem N (2010). Effects of long-chain polyunsaturated fatty acid supplementation on neurodevelopment in childhood: a review of human studies. *Prostaglandins Leukotrienes and Essential Fatty Acids*.

[15] Makrides M, Smithers LG, Gibson RA (2010). Role of long-chain polyunsaturated fatty acids in neurodevelopment and growth. *Nestle Nutrition Workshop Series: Pediatric Program*.

[16] Judge MP, Harel O, Lammi-Keefe CJ (2007). Maternal consumption of a docosahexaenoic acid-containing functional food during pregnancy: benefit for infant performance on problem-solving but not on recognition memory tasks at age 9 mo. *American Journal of Clinical Nutrition*.

[17] Malcolm CA, Hamilton R, McCulloch DL, Montgomery C, Weaver LT (2003). Scotopic electroretinogram in term infants born of mothers supplemented with docosahexaenoic acid during pregnancy. *Investigative Ophthalmology and Visual Science*.

[18] Malcolm CA, McCulloch DL, Montgomery C, Shepherd A, Weaver LT (2003). Maternal docosahexaenoic acid supplementation during pregnancy and visual evoked potential development in term infants: a double blind, prospective, randomised trial. *Archives of Disease in Childhood*.

[19] Dunstan JA, Simmer K, Dixon G, Prescott SL (2008). Cognitive assessment of children at age 2 (1/2) years after maternal fish oil supplementation in pregnancy: a randomised controlled trial. *Archives of Disease in Childhood*.

[20] Helland IB, Saugstad OD, Smith L (2001). Similar effects on infants of n-3 and n-6 fatty acids supplementation to pregnant and lactating women. *Pediatrics*.

[21] Helland IB, Smith L, Blomen B, Saarem K, Saugstad OD, Drevon CA (2008). Effect of supplementing pregnant and lactating mothers with n-3 very-long-chain fatty acids on children’s iq and body mass index at 7 years of age. *Pediatrics*.

[22] Helland IB, Smith L, Saarem K, Saugstad OD, Drevon CA (2003). Maternal supplementation with very-long-chain n-3 fatty acids during pregnancy and lactation augments children’s IQ at 4 years of age. *Pediatrics*.

[23] Tofail F, Kabir I, Hamadani JD (2006). Supplement of fish-oil and soy-oil during pregnancy and psychomotor development of infants. *Journal of Health, Population and Nutrition*.

[24] Judge MP, Harel O, Lammi-Keefe CJ (2007). A docosahexaenoic acid-functional food during pregnancy benefits infant visual acuity at four but not six months of age. *Lipids*.

[25] Teller D (1990). *Teller Acuity Card (TAC) Manual*.

[26] Saunders K, Leat SJ, Shute RH, Westall CA Visual Acuity. *Assessing Children’s Vision: A Handbook. Butterworth*.

[27] Odom JV, Bach M, Brigell M (2010). ISCEV standard for clinical visual evoked potentials (2009 update). *Documenta Ophthalmologica*.

[28] Marmor MF, Fulton AB, Holder GE, Miyake Y, Brigell M, Bach M (2009). ISCEV Standard for full-field clinical electroretinography (2008 update). *Documenta Ophthalmologica*.

[29] Fantz R, Ordy J, Udelf M (1962). Maturation of pattern vision in infants during the first six months of life. *Journal of Comparative and Physiological Psychology*.

[30] Heersema DJ, Van Hof-Van Duin J (1990). Age norms for visual acuity in toddlers using the acuity card procedure. *Clinical Vision Sciences*.

[31] Bayley N (1993). *Bayley Scales of Infant Development*.

[32] Fagan JF, Shepard PA (1991). *The Fagan Test of Infant Intelligence Manual*.

[33] Thompson LA, Fagan JF, Fulker DW (1991). Longitudinal prediction of specific cognitive abilities from infant novelty preference. *Child Development*.

[34] Wainwright PE, Xing HC, Mutsaers L, McCutcheon D, Kyle D (1997). Arachidonic acid offsets the effects on mouse brain and behavior of a diet with a low (n-6):(n-3) ratio and very high levels of docosahexaenoic acid. *Journal of Nutrition*.

[35] Willatts P (1984). The stage-IV infant’s solution of problems requiring the use of supports. *Infant Behavior and Development*.

[36] Willatts P (1984). Stages in the development of intentional search by young infants. *Developmental Psychology*.

[37] Willatts P (1999). Development of means-end behavior in young infants: pulling a support to retrieve a distant object. *Developmental Psychology*.

[38] Colombo J, Kannass KN, Shaddy DJ (2004). Maternal DHA and the development of attention in infancy and toddlerhood. *Child Development*.

[39] Griffiths R (1970). *The Abilities of Young Children. A Comprehensive System of Mental Measurement for the First Eight Years of Life*.

[40] Dunn L (1997). *Examiners Manual for the Peabody Picture Vocabulary Test*.

[41] Achenbach TM (1991). *Manual for the Child Behaviour Checklist 1.5–5 and 1991 Profile*.

[42] Lenassi E, Likar K, Stirn-Kranjc B, Brecelj J (2008). VEP maturation and visual acuity in infants and preschool children. *Documenta Ophthalmologica*.

[43] Regan D (1977). Speedy assessment of visual acuity in amblyopia by the evoked potential method. *Ophthalmologica*.

[44] Prager TC, Zou YL, Jensen CL, Fraley JK, Anderson RE, Heird WC (1999). Evaluation of methods for assessing visual function of infants. *American Association for Pediatric Ophthalmology and Strabismus*.

[45] SanGiovanni JP, Parra-Cabrera S, Colditz GA, Berkey CS, Dwyer JT (2000). Meta-analysis of dietary essential fatty acids and long-chain polyunsaturated fatty acids as they relate to visual resolution acuity in healthy preterm infants. *Pediatrics*.

[46] Clandinin MT, Van Aerde JE, Merkel KL (2005). Growth and development of preterm infants fed infant formulas containing docosahexaenoic acid and arachidonic acid. *Journal of Pediatrics*.

